# The mediating role of resilience in the relationship between perceived family social support and general life satisfaction

**DOI:** 10.3389/fpsyg.2026.1862015

**Published:** 2026-06-18

**Authors:** Mehmet Enes Sağar, Bünyamin Ateş

**Affiliations:** 1 Department of Guidance and Psychological Counseling, Education Faculty, Afyon Kocatepe University, Afyon, Türkiye; 2 Department of Guidance and Psychological Counseling, Education Faculty, Erzincan Binali Yıldırım University, Erzincan, Türkiye

**Keywords:** life satisfaction, resilience, social support, students, university

## Abstract

**Introduction:**

Life satisfaction is an important indicator of psychological well-being among university students. Family social support and resilience are considered key protective factors that may contribute to higher levels of life satisfaction. This study aimed to test a hypothesized model examining the relationships among perceived family social support, resilience, and general life satisfaction in university students.

**Methods:**

The study group consisted of 357 students (146 males, 40.9%; 211 females, 59.1%; Mage = 22.43) enrolled in various faculties of public universities in Türkiye during the 2024–2025 academic year. Data were collected online via Google Forms using the Adult Life Satisfaction Scale, the Multidimensional Scale of Perceived Social Support, the Adult Resilience Measure, and a Personal Information Form. The data were analyzed using SPSS and Mplus software.

**Results:**

The findings revealed statistically significant positive relationships between general life satisfaction and resilience, between general life satisfaction and perceived family social support, and between resilience and perceived family social support. Perceived family social support significantly and positively predicted resilience, while resilience significantly and positively predicted general life satisfaction. In addition, resilience was found to mediate the relationship between perceived family social support and general life satisfaction. The direct and indirect effects included in the model explained approximately 46% of the variance in general life satisfaction.

**Discussion:**

The findings underscore the important roles of perceived family social support and resilience in enhancing university students’ life satisfaction. The results suggest that interventions designed to strengthen family support and resilience may contribute to improving students’ psychological well-being and overall life satisfaction. Future research may further investigate these relationships across different cultural contexts and student populations.

## Introduction

Individuals’ psychological well-being occupies an important place within contemporary health criteria. The fundamental elements that contribute to happiness and provide meaning to life have been among the primary topics of interest across various disciplines for centuries. Concepts related to individuals’ positive self-evaluations—such as life satisfaction, quality of life, happiness, subjective and psychological well-being, and positive affect—not only represent indicators of individual well-being but are also considered among the core determinants of mental health and key criteria within the general health framework. Among these concepts that focus on individuals’ positive evaluations of their overall lives, general life satisfaction has emerged as a prominent research topic that has attracted increasing scholarly attention (Ateş and Sağar, 2022; Ateş and Sağar, 2024).

Life satisfaction is a subjective indicator of well-being that reflects individuals’ overall evaluations of their lives. This evaluation is formed through a comparison between individuals’ current life conditions and their personal expectations, desires, and ideals. In other words, life satisfaction can be conceptualized as a subjective perception shaped by a cognitive judgment process in which individuals assess their satisfaction with life according to their own values and standards (Neugarten et al., 1961). Life satisfaction refers to a general level of satisfaction that emerges as a result of a cognitive evaluation process based on the congruence between existing life conditions and personal expectations, ideals, and desires, encompassing individuals’ subjective evaluations of their lives as a whole (Diener, 1984, 1995; Diener et al., 1985; Diener and Lucas, 1999; Myers and Diener, 1995). In this respect, life satisfaction reflects individuals’ positive evaluations of their overall lives and their sense of contentment with life. Accordingly, this concept may be considered a criterion for understanding individuals’ overall quality of life, happiness, and satisfaction (Ateş and Sağar, 2021; Ateş and Sağar, 2022).

A review of the literature reveals evidence that life satisfaction is associated with a wide range of variables, including purpose in life and mental health (Sulandari et al., 2025), self-efficacy (Ateş and Sağar, 2022), optimism (Bhattacharyya et al., 2025), gratitude (Chauhan et al., 2025), self-esteem (Han et al., 2025), happiness and subjective vitality (Somoğlu et al., 2025), life events and coping styles (Ding et al., 2024), and self-regulation (Ateş and Sağar, 2024). In this context, life satisfaction may be regarded as a multidimensional construct that is associated with various individual, environmental, and sociodemographic variables. Examining life satisfaction in relation to different variables may contribute to reducing individuals’ psychological, emotional, and social strains and serve as an important reference point for the development of mental health intervention and support programs. Accordingly, the present study assumes that general life satisfaction may be related to perceived social support and resilience.

One of the primary variables expected to be associated with general life satisfaction in the context of this study is perceived social support. First introduced by Cassel in 1976 as a protective factor against the harmful effects of stress on health, perceived social support refers to the process through which individuals receive assistance, support, or resources aimed at enhancing their well-being via their social networks. This concept encompasses emotional, cognitive, or material assistance provided through reciprocal interactions within individuals’ social relationships (Colabianchi, 2004). In other words, perceived social support refers to accessible help and resources offered to individuals by another person, a group, or a broader community. From this perspective, perceived social support is considered a set of emotional, informational, or material resources provided by individuals’ social environments (Cohen and Syme, 1985; Lin et al., 1979).

Perceived social support can be defined as individuals’ experiences of receiving a certain level of help and solidarity through social relationships, such as those with family members. It consists of two main components: structural and functional. The structural component includes quantitative aspects such as the number of individuals in one’s social network, the frequency of interactions, and the intensity of social contacts. The functional component refers to individuals’ perceptions and satisfaction regarding the qualitative contributions obtained from social relationships, including emotional support (e.g., empathy and care), informational support (e.g., guidance and advice), appraisal support (e.g., feedback in decision-making processes), and instrumental support (e.g., financial or practical assistance) (Finlayson et al., 2025). Perceived social support reflects individuals’ perceptions of the assistance and support provided by various sources within their social environment and their beliefs regarding the continuity of this support. Because it is based on subjective evaluations, individuals’ perceptions of being supported are considered more salient than the actual amount of support received. Sources of perceived social support may include family members, friends, teachers, spouses, and other significant individuals (Sağar, 2022).

Within the scope of the present study, perceived family social support—one of the sources of perceived social support—is examined. This concept refers to individuals’ subjective perceptions of the emotional, informational, instrumental, and appraisal support provided by family members, as well as their beliefs regarding the reliability of this support. Perceived social support, reflecting the care and assistance individuals feel they receive from others and their families, plays a critical role in enhancing individuals’ positive mental states and contributes to mental resilience by helping them cope with difficult situations and adversity (Mai et al., 2021). From another perspective, perceived social support functions as a protective factor that helps alleviate psychological distress during stressful life events and enhances individuals’ resilience in such processes (Richmond et al., 2007). Accordingly, perceived family social support may be considered to be related to resilience. Indeed, research on orphaned children and those experiencing family loss has shown that a lack of perceived social support leads to internalized problems (e.g., anxiety, depression, and low self-esteem) as well as externalized problems such as dysfunctional behaviors (Makame et al., 2002). Studies in the literature further indicate a relationship between perceived social support and resilience (Asghar, 2025; Siswadi et al., 2023). Moreover, resilience has been conceptualized in relation to positive adaptation to adversity, psychological well-being, flexibility, strength, healthy social networks, and satisfaction with perceived social support (Dias et al., 2015). In this respect, it may be suggested that perceived family social support facilitates the development of more resilient and effective coping attitudes in the face of stressful and challenging life events, thereby positively influencing resilience levels by increasing individuals’ tendencies to evaluate their lives more positively.

Another key variable expected to be associated with general life satisfaction in this study is resilience. The concept of resilience refers to individuals’ capacity to remain resilient in the face of stressful life events and psychological risk factors, to cope effectively with adversity, and to maintain balance and harmony in life by sustaining psychosocial functioning (Wagnild and Young, 1990, 1993). In other words, resilience is defined as individuals’ ability to adapt to challenging life conditions, maintain functioning, preserve mental health, or reconstruct it when disrupted (Rutter, 1987; Wald et al., 2006; Zautra, 2009). Resilience is considered a multidimensional capacity that encompasses not only individuals’ ability to cope with stress and adapt to adverse conditions at individual or environmental levels but also their potential to experience long-term life satisfaction (Masten and Barnes, 2018; Robbins et al., 2018).

Resilience reflects individuals’ ability to cope effectively with stress-inducing situations and psychological risk factors while maintaining adaptation to their environment. It is also associated with positive psychological characteristics such as functional behavior, stress management, flexibility, recovery, and hope for the future. The ability to adapt following negative life experiences, demonstrate emotional endurance, and sustain general life satisfaction through such processes constitutes a fundamental indicator of resilience (Bonanno, 2004; Masten and Barnes, 2018; Wagnild and Young, 1990, 1993). Research suggests that resilience is related to general life satisfaction in the context of positively evaluating life and experiencing happiness. For instance, Rahman et al. (2025) emphasize that individuals who are more satisfied with their lives are more likely to cope effectively with stress and adapt successfully. Furthermore, evidence in the literature supports the association between resilience and general life satisfaction (Nadeem et al., 2025; Yan et al., 2022). Accordingly, individuals’ abilities to struggle with challenging life situations, demonstrate resilience, and employ effective coping strategies may increase their tendencies to evaluate their lives more positively, thereby enhancing general life satisfaction.

General life satisfaction may be defined as individuals’ positive evaluations of their lives as a whole, their sense of contentment with life, and the fulfillment they derive from it. In this regard, individuals who hold a positive perspective toward their lives are expected to experience higher levels of general life satisfaction. When considered specifically among young individuals such as university students, those who approach life with a positive outlook may be expected to demonstrate higher levels of general life satisfaction. Young individuals who develop positive life perceptions and achieve high levels of general life satisfaction have the potential to contribute positively to both themselves and their social environments. Although studies in the literature have examined the relationships between general life satisfaction and various individual and environmental variables across different age groups, research simultaneously addressing perceived family social support and resilience alongside general life satisfaction remains limited. In this respect, the present study is considered important in terms of contributing to the literature and providing a foundation for preventive and intervention-based mental health practices aimed at enhancing general life satisfaction among young individuals. Accordingly, the study aims to test a hypothesized model examining the relationships among perceived family social support, resilience, and general life satisfaction.

## Method

### Research model

In the present study, a relational survey model was employed. The relational survey model is designed to determine the existence and/or degree of covariation among variables (Karasar, 2016; Creswell, 2014).

### Study group

The study group consisted of 357 students (146 male, 40.9%; 211 female, 59.1%) enrolled in various faculties of public universities in Türkiye during the 2024–2025 academic year. The distribution of the study group by gender is presented in Table 1. In addition, the mean age of the participants was determined to be 22.43.

**Table 1:** Distribution of the study group by gender.

Gender	N	%
Male	146	40.9
Female	211	59.1
Total	357	100

### Data collection instruments

#### Adult life satisfaction scale

The scale was developed by Kaba et al. (2018). It consists of 21 items rated on a five-point Likert scale and includes five subdimensions: general life satisfaction, relationship satisfaction, self-satisfaction, social environment satisfaction, and job satisfaction. The Cronbach’s alpha internal consistency coefficient of the scale was reported as 0.89; for the subdimensions, it was 0.84 for general life satisfaction, 0.84 for relationship satisfaction, 0.77 for self-satisfaction, 0.73 for social environment satisfaction, and 0.86 for job satisfaction. In the present study, only the general life satisfaction subdimension was used as a data collection instrument. Within the scope of this study, the overall Cronbach’s alpha internal consistency coefficient of the scale was calculated as 0.87, and 0.84 for the general life satisfaction subdimension.

#### Multidimensional scale of perceived social support

The scale was developed by Zimet et al. (1988)and adapted into Turkish by Eker et al. (2001). It consists of 12 items rated on a seven-point Likert scale and includes three subdimensions: family, friends, and significant other. The Cronbach’s alpha internal consistency coefficient of the scale was reported as 0.89; for the subdimensions, it was 0.85 for family support, 0.88 for friend support, and 0.92 for significant other support. In the present study, the family subdimension of the scale was used as the data collection instrument. Within the scope of this study, the overall Cronbach’s alpha internal consistency coefficient of the scale was calculated as 0.89, and 0.82 for the family subdimension.

#### Adult resilience measure

The scale was adapted into Turkish by Arslan (2015). It consists of 21 items rated on a five-point Likert scale and includes four dimensions: relational disruptions, individual resources, cultural-contextual resources, and familial resources. The total Cronbach’s alpha internal consistency coefficient of the scale was reported as 0.94. Within the scope of the present study, the total Cronbach’s alpha internal consistency coefficient of the scale was calculated as 0.92.

#### Personal information form

The personal information form was developed within the scope of this study in accordance with the principle of confidentiality in order to obtain personal information from the university students who constituted the study group.

### Data collection and analysis

In this study, data were collected online via Google Forms. The data collection instruments prepared through Google Forms were sent via e-mail to university students enrolled in public universities located in different regions of Türkiye, and students were invited to participate in the study. The research was conducted in line with the principle of confidentiality, and informed consent was obtained from all participants after they were informed about the study. Participants were required to read and electronically approve the informed consent form before accessing the survey questions; therefore, all 357 participants included in the study provided informed consent. The online data collection process lasted approximately 1 week.

Prior to data analysis, assumptions regarding missing data, outliers, normality, linearity, homogeneity, and multicollinearity were examined sequentially. Missing data analysis revealed that there were no missing values in the dataset. For univariate normality, standardized z scores (±3), skewness and kurtosis values (±1), and histogram plots were examined. To identify outliers that might violate the assumptions of multivariate normality and linearity, Mahalanobis distance, Cook’s distance, leverage values, scatterplots, and correlation coefficients were examined. The analyses indicated that the datasets met the assumptions of univariate normality, multivariate normality, and linearity.

When examining multicollinearity among variables, it was determined that there were no correlation coefficients above 0.80, tolerance values were greater than 0.20, variance inflation factor (VIF) values were below 10, and condition index (CI) values were below 30. Accordingly, no multicollinearity problem was detected among the variables (Akbulut, 2010; Büyüköztürk, 2011; Field, 2013; Tabachnick and Fidell, 2013).

The relationships among the variables were examined using the Pearson product–moment correlation coefficient. A hypothesized model was proposed to examine the relationships among the variables. The hypothesized model was analyzed using structural equation modeling (SEM). To determine the goodness of fit of the models obtained through SEM, chi-square values (*χ*^2^), RMSEA, SRMR, CFI, and TLI indices were used (Brown, 2015). In this study, indirect (mediating) effects and standard error values were calculated using 5,000 bootstrap samples at a 95% confidence interval. All analyses were conducted using IBM SPSS Statistics 27.00 and Mplus 8.3.

### Ethical approval

Ethical approval for the study was obtained from the Afyon Kocatepe University Social and Humanities Scientific Research and Publication Ethics Committee (Decision Date: 22.03.2023; Meeting No: 03; Document No: 172785; Decision No: 2023/82).

## Results

In this section, the analysis results regarding university students’ general life satisfaction, perceived family social support, and resilience scores are presented. The means and standard deviations of the study group are presented in Table 2.

**Table 2:** Means and standard deviations.

Variable	N	M	SD
General life satisfaction (G.L.S.)	357	20.06	3.31
Resilience (R.)	357	82.28	12.31
Perceived family social support (P.F.S.S.)	357	19.96	5.40

As shown in Table 2, the mean scores of general life satisfaction (M = 20.06; SD = 3.31), resilience (M = 82.28; SD = 12.31), and perceived family social support (M = 19.96; SD = 5.40) were determined. The correlation results for the study variables are presented in Table 3.

**Table 3:** Correlation coefficients among the study variables.

Variable	G.L.S.	R.	P.F.S.S.
General life satisfaction (G.L.S.)	1		
Resilience (R.)	0.590^**^	0.1	
Perceived family social support (P.F.S.S.)	0.345^**^	0.515^**^	0.1

**p < 0.01.

According to Table 3, there were statistically significant positive correlations between general life satisfaction and resilience (*r* = 0.590, *p* < 0.01), between general life satisfaction and perceived family social support (*r* = 0.345, *p* < 0.01), and between resilience and perceived family social support (*r* = 0.515, *p* < 0.01).

### Measurement model

Before testing the hypothesized model, the measurement model was examined. The goodness-of-fit indices of the measurement model were found to be *χ*^2^(74) = 229.373, *χ*^2^/df = 3.09, RMSEA = 0.077 (90% CI [0.066, 0.088]), CFI = 0.941, TLI = 0.927, and SRMR = 0.053. These fit indices indicated that the measurement model demonstrated an acceptable level of fit. Since the measurement model exhibited adequate fit, the structural model was subsequently tested (Kline, 2011).

### Structural model

Following the establishment of adequate fit indices for the measurement model, the structural model was tested. The goodness-of-fit indices for the structural model were found to be *χ*^2^(74) = 229.373, *χ*^2^/df = 3.09, RMSEA = 0.077 (90% CI [0.066, 0.088]), CFI = 0.941, TLI = 0.927, and SRMR = 0.053. Although the overall fit indices of the model were within acceptable ranges, the standardized beta coefficient for the path from perceived family social support to general life satisfaction was found to be low and statistically non-significant (*β* = −0.087, 95% CI [−0.241, 0.066], t = −1.112, *p* < 0.05). Based on this finding, it was decided to constrain the direct path from perceived family social support to general life satisfaction to zero (i.e., to remove it from the model). Accordingly, the final model was constructed by taking the theoretical framework into consideration.

To determine the most appropriate model between the hypothesized model and the final model, the Bayesian Information Criterion (BIC) was used. A decrease of two or more units in the BIC value is considered an appropriate criterion for model selection (Raftery, 1995). In the hypothesized model, the BIC value was 16580.624, whereas constraining the direct path from perceived family social support to general life satisfaction to zero (in the final model) resulted in a BIC value of 16576.483, indicating a decrease of approximately four units. Therefore, the final model was determined to be more appropriate.

**Figure 1:**
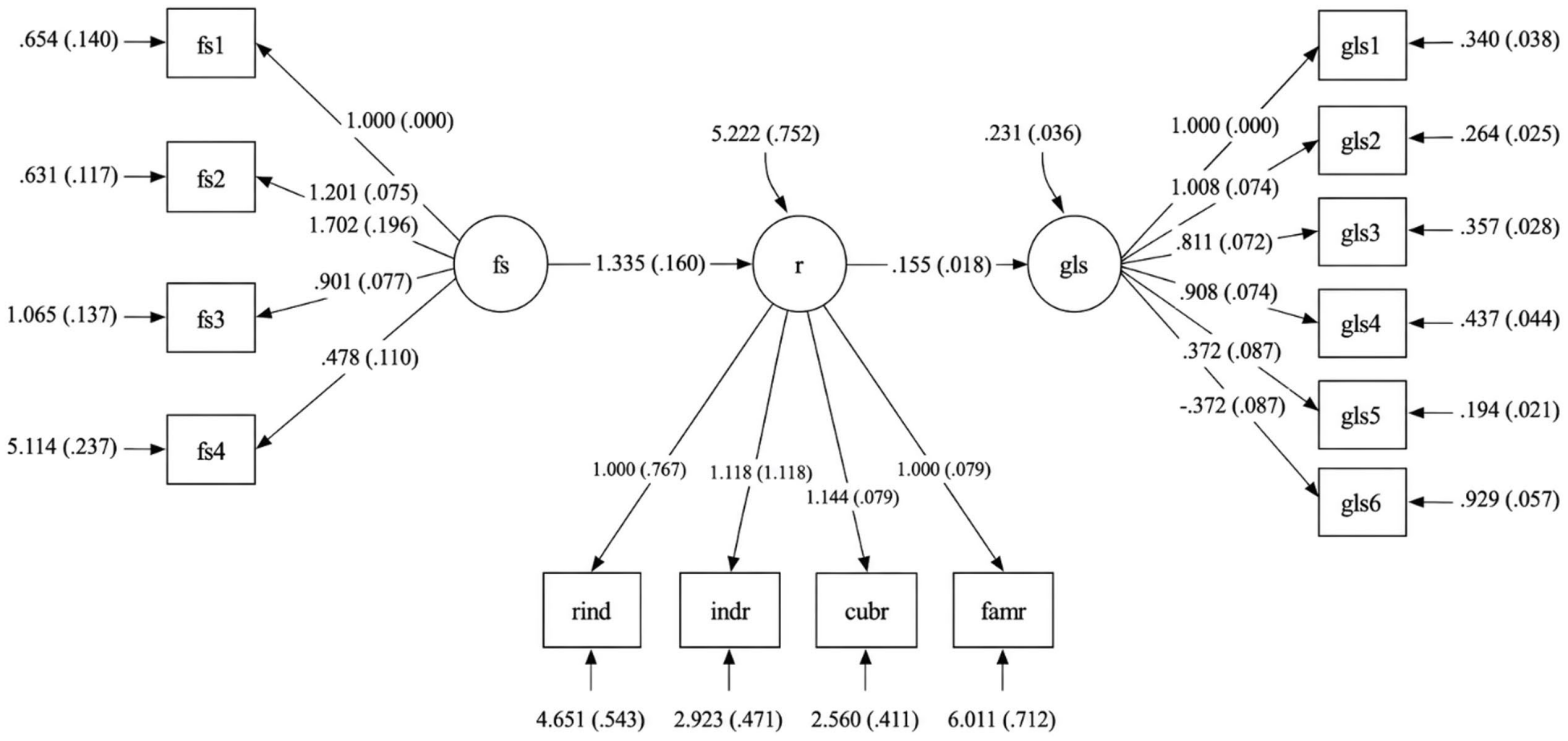
Structural equation modeling of the final model.

After constraining (i.e., removing) the direct path from perceived family social support to general life satisfaction, partial improvements in model fit indices were observed. The fit indices for the final model were *χ*^2^(75) = 231.111, *χ*^2^/df = 3.08, RMSEA = 0.076 (90% CI [0.065, 0.088]), CFI = 0.940, TLI = 0.927, and SRMR = 0.053, indicating that the final model demonstrated an adequate level of fit. The results of the final model are presented in Figure 1.

In the final model, the statistical significance of the relationships among the latent variables was interpreted based on t values and standardized beta coefficients. The results related to these values are presented in Table 4.

**Table 4:** Standardized direct effects and t values for the final model.

	B	*β*	S.E	*t*
PFSS→R	1.335	0.606	0.047	12.92^**^
R→GLS	0.155	0.680	0.047	14.36^**^

**p < 0.01.

According to Table 4, the direct effects of perceived family social support on resilience and of resilience on general life satisfaction were examined. The results indicated that perceived family social support significantly and positively predicted resilience (*β* = 0.606, 95% CI [0.514, 0.698], *t* = 12.92, *p* < 0.01). In addition, resilience was found to significantly and positively predict general life satisfaction (*β* = 0.680, 95% CI [0.587, 0.772], *t* = 14.36, *p* < 0.01).

In the present study, indirect (mediating) effects were examined in addition to direct effects. To test the mediating role (indirect effect) of resilience in the model, a bootstrapping procedure with 5,000 resamples was conducted using a 95% confidence interval. Through this procedure, indirect effect coefficients and standard error values were calculated (see Table 5). The absence of zero within the calculated confidence intervals (CI) indicates that the indirect (mediating) effect is statistically significant.

**Table 5:** Indirect effect values of the variables.

	*β*	S.E	*t*	%95 CI
PFSS R GLS	0.412	0.044	9.36^**^	[0.326–0.498]

**p < 0.01.

According to Table 5, examination of the indirect effects indicates that perceived social support significantly predicts general life satisfaction through resilience (*β* = 0.412, 95% CI [0.326, 0.498], *p* < 0.01). The absence of zero within the calculated confidence intervals (CI) demonstrates that the mediating (indirect) effect of resilience in the model is statistically significant. Overall, the direct and indirect effects of all variables included in the model account for approximately 46% of the variance in general life satisfaction (*R*^2^ = 0.462).

## Discussion, conclusion and recommendations

The present study aimed to test a hypothesized model examining the relationships among perceived family social support, psychological resilience, and general life satisfaction. The results indicated that there were significant positive relationships between general life satisfaction and psychological resilience, between general life satisfaction and perceived family social support, and between psychological resilience and perceived family social support. In addition, perceived family social support was found to positively and significantly predict psychological resilience, while psychological resilience positively and significantly predicted general life satisfaction. Furthermore, perceived family social support was found to significantly predict general life satisfaction through the mediating role of psychological resilience. The direct and indirect effects of all variables in the model collectively explained approximately 46% of the variance in general life satisfaction. An examination of the structural model results revealed that the hypothesized model was supported. The direct and indirect effects identified in this model may contribute to a better understanding of the factors influencing general life satisfaction.

From a theoretical perspective, the findings of the present study may be interpreted within the framework of positive psychology and resilience theory. Positive psychology emphasizes the role of protective individual and environmental factors in promoting well-being, psychological adjustment, and life satisfaction (Seligman and Csikszentmihalyi, 2014). In this context, perceived family social support can be considered an important environmental resource that strengthens individuals’ coping capacities and facilitates adaptive psychological functioning. Similarly, resilience theory conceptualizes resilience as a dynamic process through which individuals maintain or regain psychological well-being despite adversity and stressful experiences (Masten, 2001; Rutter, 1987). The findings of the present study support these theoretical assumptions by demonstrating that perceived family social support contributes to life satisfaction indirectly through enhancing psychological resilience. Accordingly, family support may function not only as a direct emotional resource but also as a developmental mechanism that enables university students to cope more effectively with stressors encountered during emerging adulthood.

Life satisfaction is an important psychological construct that refers to individuals’ overall level of satisfaction with life and the extent to which they evaluate their lives positively. Achieving life satisfaction plays a critical role in individuals’ ability to organize their lives, effectively fulfill their roles and responsibilities, and develop a positive perspective toward life. When considered specifically within the context of university students, this process necessitates not only positive academic development but also growth in social and personal domains. University students are expected not only to focus on academic achievement but also to cultivate positive attitudes toward life and adopt lifestyles that enhance their life satisfaction. Students’ ability to experience satisfaction in their current lives as well as in their future social and professional roles is directly related to how they evaluate their lives. In this context, perceiving one’s life within a positive framework can be considered a functional factor that directly contributes to life satisfaction. Positive evaluations of life among university students may be regarded as an indicator of high life satisfaction. Such evaluations reflect students’ general sense of fulfillment with life and their positive perceptions, which may also signal potential success across various life domains. Therefore, developing motivational and supportive interventions aimed at increasing university students’ life satisfaction is of great importance. Identifying variables associated with students’ life satisfaction, raising awareness in this area, and contributing to the existing body of knowledge may provide significant benefits in enhancing university students’ quality of life and overall well-being. Accordingly, scientific studies conducted in this field may enable a better understanding of the dynamics of university life, facilitate the development of more effective solutions to student needs, and support the establishment of sustainable support systems. For these reasons, such research is considered valuable from both theoretical and practical perspectives (Ateş and Sağar, 2021; Ateş and Sağar, 2022; Ateş and Sağar, 2024). Within this framework, the findings of the present study were discussed in light of the relevant literature.

The first finding of the study revealed that general life satisfaction and perceived family social support were positively and significantly related, and that psychological resilience played a mediating role in the relationship between general life satisfaction and perceived family social support. A review of the literature indicated that there are no direct studies specifically examining the relationship between general life satisfaction and perceived family social support. However, existing studies demonstrating a significant positive relationship between life satisfaction and perceived social support support the findings of the present study (Ahmed et al., 2025; González et al., 2025; Yıldırım et al., 2025). Further examination of the literature regarding the mediating role of psychological resilience in the relationship between general life satisfaction and perceived family social support revealed that although no studies directly addressing this specific mediation were found, several studies support the mediating role of psychological resilience in the relationship between life satisfaction and perceived social support. In particular, findings reported by Wu et al. (2022); Sevari et al. (2020); as well as Yang et al. (2022); Cao and Zhou (2021); and Guo (2017) are consistent with the results of the present study, indicating that psychological resilience serves as a mediator between life satisfaction and perceived social support.

Beyond supporting previous empirical findings, the present results may also reflect the developmental characteristics of emerging adulthood. University students frequently encounter academic stress, uncertainty regarding future careers, financial difficulties, and adaptation challenges associated with increased independence. During this developmental period, family support may provide emotional security and psychological stability that foster resilient coping mechanisms. In turn, resilient students may be more capable of interpreting stressful experiences as manageable rather than overwhelming, thereby maintaining higher levels of life satisfaction. In this respect, resilience appears to operate as an internal psychological resource translating external social support into positive life evaluations. This finding is also compatible with ecological and developmental perspectives suggesting that supportive family environments strengthen adaptive capacities and promote well-being outcomes among young adults (Bronfenbrenner, 1979; Masten, 2014).

Another important finding of the study was that general life satisfaction and psychological resilience were positively and significantly related among university students. In other words, as students’ levels of psychological resilience increase, their levels of general life satisfaction also tend to increase. A review of the literature revealed that this finding is consistent with previous research reporting significant positive relationships between life satisfaction and psychological resilience (González et al., 2025; Rahman et al., 2025; Yan et al., 2022; Zhang et al., 2022). University students with high levels of psychological resilience are better able to cope flexibly with stress, difficulties, trauma, and adverse life events, and they possess a greater capacity to recover from such challenges. As a result, these students may evaluate their lives more positively, perceive their lives as meaningful and satisfying, and consequently experience higher levels of general life satisfaction. In this regard, psychological resilience is considered to play a functional role in enhancing university students’ general life satisfaction.

The strong predictive role of resilience identified in the present study also suggests that resilience may function as a central protective mechanism in university students’ psychological adjustment processes. University life is characterized by continuous developmental transitions and psychosocial demands. Students with higher resilience may possess greater emotional regulation skills, cognitive flexibility, and problem-solving abilities, enabling them to maintain psychological balance under stress. This interpretation is consistent with previous studies emphasizing that resilience contributes to adaptive functioning, emotional well-being, and long-term psychological adjustment (Bonanno, 2004; Masten and Barnes, 2018). Therefore, resilience-focused interventions implemented within university counseling services may contribute not only to reducing psychological difficulties but also to strengthening students’ overall life satisfaction and well-being.

Another noteworthy finding of the present study is that the direct effect of perceived family social support on general life satisfaction became statistically non-significant when resilience was included in the structural model. This result may indicate that the influence of family support on life satisfaction operates primarily through psychological resilience rather than through a direct pathway. In other words, family support may enhance students’ abilities to cope with adversity, regulate emotions, and adapt to challenging life experiences, and these strengthened resilience capacities may subsequently increase life satisfaction. This finding contributes to the literature by clarifying the underlying psychological mechanism linking family support and life satisfaction. Moreover, it highlights the importance of considering resilience as a key explanatory variable in studies examining the relationship between social support and well-being.

From a practical perspective, the findings of the present study provide important implications for mental health professionals, university counselors, educators, and policymakers working with university students. The results indicate that strengthening perceived family social support and psychological resilience may contribute significantly to increasing students’ general life satisfaction and psychological well-being. In this regard, university counseling centers may develop psychoeducational and preventive intervention programs focusing on resilience enhancement, stress management, emotional regulation, coping skills, and problem-solving strategies. In addition, interventions designed to strengthen family communication and supportive family relationships may indirectly improve students’ life satisfaction by fostering resilience capacities. Universities and higher education institutions may also benefit from implementing student support systems that promote social connectedness, mentoring opportunities, and accessible psychological counseling services. From a policy perspective, the findings suggest that mental health policies targeting university students should not only focus on reducing psychological problems but also prioritize the development of protective psychosocial resources such as family support and resilience. Accordingly, integrating resilience-based mental health practices and family-oriented support approaches into higher education policies may contribute to improving students’ overall well-being, academic adjustment, and quality of life. Despite the contributions of the present study, several limitations should be acknowledged. First, the study employed a cross-sectional research design; therefore, causal inferences cannot be drawn regarding the relationships among perceived family social support, resilience, and general life satisfaction. Second, all data were collected through self-report measures, which may increase the risk of common method bias and social desirability effects. Third, the study sample consisted exclusively of university students enrolled in public universities in Türkiye. Therefore, the generalizability of the findings to other age groups, cultural contexts, or educational settings should be considered with caution.

Future studies employing longitudinal designs, multiple data sources, and more diverse samples may provide a more comprehensive understanding of the relationships among these variables.

Future studies may further strengthen the literature by examining the relationships among perceived family social support, psychological resilience, and life satisfaction within different sociocultural and contextual frameworks. In particular, comparative studies involving students from different cultural backgrounds, socioeconomic levels, or educational settings may provide a deeper understanding of how contextual factors influence these variables. In addition, future research may benefit from investigating technology-based and contemporary factors—such as digital social support, social media use, loneliness in online environments, and artificial intelligence–assisted counseling applications—in relation to resilience and life satisfaction among university students. Longitudinal and experience-sampling studies may also contribute to understanding how resilience and perceived support dynamically change during critical developmental and academic transition periods. Furthermore, integrating qualitative approaches may provide richer insights into university students’ lived experiences regarding family support, resilience, and well-being.

In conclusion, the present study demonstrated that perceived family social support contributes to university students’ general life satisfaction through the mediating role of psychological resilience. The findings emphasize that resilience is not only an individual psychological strength but also a mechanism shaped by supportive social relationships, particularly family support. In this respect, strengthening both family support systems and resilience capacities may play a critical role in promoting university students’ psychological well-being and life satisfaction. The study also contributes to the existing literature by offering a theoretically grounded model explaining how family support and resilience jointly influence life satisfaction among university students.

## Data Availability

The original contributions presented in the study are included in the article/supplementary material, further inquiries can be directed to the corresponding author.
